# Hollow viscus injury in children: Starship Hospital experience

**DOI:** 10.1186/1749-7922-2-14

**Published:** 2007-06-04

**Authors:** Saleh M Abbas, Vipul Upadhyay

**Affiliations:** 1 University of Auckland; Starship Children's Hospital, Auckland, New Zealand

## Abstract

Starship Children's Hospital in Auckland, New Zealand, serves a population of 1.2 million people and is a tertiary institution for pediatric trauma. This study is designed to review all cases of abdominal injury (blunt and penetrating) that resulted in injury of a hollow abdominal viscus including the stomach, duodenum, small intestine, large intestine and urinary bladder. The mechanism of injury; diagnosis and outcome were studied. This was done by retrospective chart review of patients admitted from January 1995 to December 2001. Thirty two injuries were found in 29 children. The age ranged from 7 months to 15 years with boys represented more commonly. Small bowel was the most frequently injured hollow viscus. Computerized Tomography (CT scan) is an extremely useful tool for the diagnosis of HVI.

## Background

Trauma is the leading cause of morbidity and mortality in childhood with motor vehicle accidents causing most childhood deaths (*ATLS 1997*) [1].

Perforation of the gastrointestinal tract is relatively infrequent sequel of blunt abdominal trauma. Incidence of hollow visceral injury varies from <1%–8.5% [2,3] Diagnostic delay is associated with increased morbidity and hospital stay and perhaps increased mortality especially when there is associated severe head injury [4].

This study is designed to review all cases of abdominal injury (blunt and penetrating) that resulted in injury of a hollow abdominal viscus including the stomach, duodenum, small intestine, large intestine and urinary bladder. English literature was reviewed for the purpose of this study.

## Methods

We retrospectively reviewed all cases of hollow viscus injuries that were admitted to Starship hospital over 6 year period from January 1995 to December 2001.

The hospital database was searched using ICD-9 and ICD-10 codes for abdominal trauma, stomach injury, duodenal injury, jejunal injury, ileal injury, urinary bladder injury, colon injury with specification of the site as caecum ascending colon, transverse colon, descending colon, sigmoid colon and rectum.

The patient charts were reviewed for demographic data, type of injury, mechanism of injury, symptoms at presentation, radiology, methods of diagnosis, treatment, hospital stay, associated injuries and complications.

## Results

**Figure 1:**
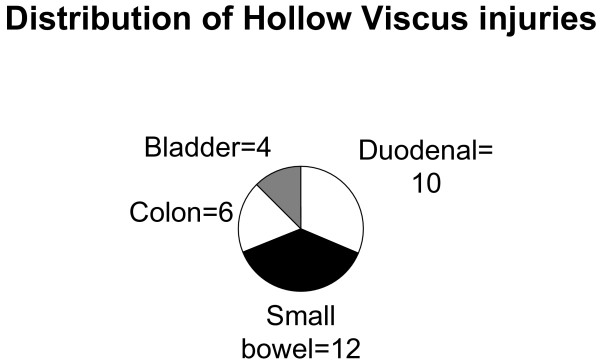
Distribution of HVI.

Thirty-two hollow visceral injuries were identified in 29 children. Median age was 9 years (range 7 months – 15 years); Twenty-one male and 8 females. The median length of stay was 10 days (range 4–23; 95% CI 7–12). There was one death, 3 injuries were non accidental one of them was child abuse and two were assaults. One of the assaults was by a child of same age and the other was by an intruder who attacked an adult and injured the child, who was in the lap of the adult. This is the reason why we have not called all three non-accidental injuries as child abuse. Figure 1 shows the distribution of HVI.

### Duodenal injuries

Ten children had duodenal injuries 5 boys and 5 girls, median age 9 (range 6–14). Eight had duodenal hematoma (median age 9), involving the second part in all patients, and extended to the 3^rd ^part in 2 patients. The mechanisms of all duodenal injuries were handle bar injury in 2, motor vehicle accident (MVA) in 3 (median age 10), fall from height on a hard object in 4 (median age 6); one was kicked by a horse (11 years old). Two patients had perforation of the second part of the duodenum one resulted from MVA (age 12) and in one child from fall from height on a hard object (4 years old). Duodenal injuries in children involved in MVA were thought related to seat belt; all children involved in MVA were backseat passengers with lap belt buckled in.

All patients presented with abdominal pain and epigastric tenderness. Both handle bar injuries were associated with bruises in the epigastric region; one of the MVA patients had lap seat belt marks of bruises on the anterior abdominal wall and was found to have first Lumbar vertebral (L1) compression fracture. All patients who had MVA had associated injuries which included head injury [2], L1 compression fracture, rupture spleen [1], fracture femur [2], Right Kidney [2], one patient had multiple fractures. All were haemo-dynamically stable except one who had multiple fractures.

The diagnosis of duodenal hematoma was made in 8 patients by CT scan; one had ultrasound scan (USS) and upper gastro-intestinal (UGI) series as a diagnostic method, median age of those children was 10. CT showed free air in both cases that had perforation; theses were4 and 10 years old.

The two patients with duodenal perforation had emergency laparotomy and closure of the perforation with an omental patch followed by total parenteral nutrition (TPN) which was initiated due to delayed recovery from associated injuries and prolonged ileus; TPN was used for 10 days in one patient and 14 days in the second one. No major complications were seen and the average hospital stay was12.2 days. There were no deaths.

### Small bowel injuries

Twelve patients were diagnosed with small bowel injury (perforation). Eleven males and one female with median age of 8 year (range 2–15 years). Eight had jejunal perforations; most of them were within 40 cm from the duodeno-jejunal (DJ) Flexure.

All except one were on the anti-mesenteric border. Five were related to MVA, one handle bar injury, one fall from height, and one was related to child abuse that had subdural hematoma and had presented with abdominal distention and persistent vomiting. There were 3 ileal perforations; 2 were related to MVA and one patient was kicked by a horse. There was one Meckel's diverticulum perforation. MVA cases presented with acute abdominal symptoms and signs. Six out of the 7 cases has lap seat belt bruises, the handle bar injury and the horse kick cases had bruises on the anterior abdominal wall.

Seven patients had CT scans, 5 found to have free air on CT and taken to theatre, 2 had CT features suggestive of small bowel injury but no free air, the diagnosis was delayed in both, one developed peritonitis after 48 hours and was taken to theatre the second developed abdominal abscess and had laparotomy after 11 days, both patients had clinical features of acute abdomen and seat belt signs. The 5 patients who did not have CT scan were dealt with on clinical assessment, 2 had plain films that showed free air, 2 had clinical evidence of peritonitis and one was unstable with multiple injuries and was taken to theatre. Jejunal injuries were dealt with by closure of the perforation except one patient who had complete transection of the jejunum near the DJ flexure and needed end-to-end anastomosis. Ileal perforations were closed and the injury related to child abuse was treated with resection and end-to-end anastomosis due to unhealthy margins. Apart from the 2 cases that had delayed diagnosis the mean time between admission and operation was 6 hours.

All patients made uneventful recovery with median hospital stay of 9 days (range 5–23) 2 developed wound infections; no one had long term complications.

### Colonic injuries

Six patients had colonic injuries, 5 males and one female; with median age 14 (range 5–16). Three had hematoma of the right colon and caecum, 2 caused by MVA of which one was treated conservatively and one sustained by knee to the abdomen during cricket match which needed right hemicolectomy. All were diagnosed on CT scan. 2 had stab wound injuries to the descending colon and sigmoid colon both were treated by resection and end to end anastomosis. The sixth (9 year old female) patient had severe multiple injuries including aortic dissection, fracture L3 spinal cord injury, head injury and perforation of the right colon treated with right hemicolectomy and ileostomy she died after 19 days from multiple organ failure related to sepsis.; the rest recovered well 2 of them were readmitted 2 years later with partial small bowel obstruction which resolved without operative treatment.

### Urinary bladder injuries

Four urinary bladder injuries were found 2 males and 2 females. Two were caused by MVA and 2 by car versus pedestrian. All cases had frank hematuria. Three had pelvic fractures and one had bruises around the pelvic bones.

All were diagnosed by CT and showed leak of the contrast outside the bladder. Two injuries were intra-peritoneal and treated with laparotomy and repair of the bladder and 2 were extra-peritoneal treated with indwelling catheter for ten days.

### Stomach

There was one stomach injury caused by penetrating stab wound that caused penetration of the colon, liver, spleen and diaphragm. The patient was 7 months old, he needed laparotomy and repair of the perforations and packing of the liver and spleen, later on developed liver abscess and eventually recovered.

### Associated injuries: Table 3

Twelve patients had associated injuries, 6 of the duodenal injury patients had associated injuries; one of the small bowel injury patient had other injury. All bladder injuries were associated with other injuries, 3 pelvic fractures, and 2 severe head injuries.

### Imaging

CT scan was done in most patients who were admitted with significant abdominal injuries. The positive yield is high especially when it was combined with clinical findings, of the 10 duodenal injuries 9 had CT scans, all showed the correct diagnosis and revealed other associated injuries.

Out of the 7 patients who had CT scan for suspected perforation 2 were misdiagnosed. Both had radiological signs suggestive of small bowel injury and clinical signs of acute abdomen, however they were managed conservatively due to absence of free air, both developed complication (one had peritonitis, the second developed intra-abdominal abscess) and needed laparotomy; eventually both recovered well.

Abdominal ultrasound in not routinely obtained for blunt abdominal injury at this hospital, one child had abdominal ultrasound initially; due to unusual presentation (child abuse).

### Seat belt injuries

Most patients (6 out of 7) who had small bowel perforation and were involved in MVA had seat belt marks on their abdomen. It seems that with a mechanism like MVA and presentation with seat belt marks on the abdomen should raise a very high suspicion of HVI.

### Child abuse

We identified one patient with perforation of the distal jejunum. He was 21 month old who was previously admitted with subdural hematoma presented on this occasion with abdominal distention, vomiting and bruises on the anterior abdominal wall and scrotal bruises and altered liver function test. Abdominal ultrasound exam (done before surgical involvement) showed large amount of free fluid in the abdominal cavity, CT scan was then obtained and showed free air in the peritoneal cavity. At laparotomy there was jejunal perforation and a defect in the mesentery. A delayed perforation had probably occurred due to mesenteric vascular injury and ischaemia of the bowel wall, the increased liver enzymes were caused by paracetamol poisoning (part of child abuse). The child has recovered well from surgery.

## Discussion

The incidence and pattern of blunt abdominal trauma has not changed over the last decade. Since the introduction of motor vehicle safety measures mainly the lap seat belt, this pattern of trauma is mainly due to the reduction in mortality related to head injury. However the lap seatbelt has increased the incidence of mesenteric and small bowel injuries.

Diagnosis of Hollow visceral injuries in blunt abdominal trauma presents a significant challenge to the trauma team. Although it is relatively easy to establish the diagnosis in clinically acute abdomen the minimally symptomatic patient requires a more aggressive approach to establish the diagnosis.

Conservative management of blunt abdominal trauma may increase the risk of delay in the diagnosis of hollow viscus injuries. Despite the clinical suspicion, diagnosis of hollow viscus injury is often delayed in children. This is especially when there is associated head injury, spinal injury and solid organ abdominal injuries. This is largely due to its contribution in altering peritoneal signs [5].

CT scan is the investigation of choice in the haemo-dynamically stable patient [6].

Jamieson et al in a series of 34 CT studies in blunt abdominal trauma concluded that CT had 100% positive predictive value for bowel perforation when extra-luminal air, free fluid, wall thickening, bowel wall enhancement and bowel dilatation are present on CT [7].

Clinical suspicion combined with CT scan of the abdomen is presently the most sensitive combination for the diagnosis of visceral injury [8]. Plain abdominal film was obtained as a part of the trauma assessment included with imaging of the pelvis; its value was to show free gas which was evident in four cases. The main cause of death seems to be associated injuries; mainly head injury [9] or multiple injuries as in our patient.

Abdominal US scan is not used in this institute for abdominal injury. Stengel et al has conducted a meta-analysis on 30 trials of US scan in abdominal trauma and concluded that it has very low sensitivity despite high specificity of both free fluid and organ injury and does not preclude the need for CT scanning [10].

Although uncommon, blunt duodenal injury is often misdiagnosed with increased complications [11]. In our series only one patient had delayed diagnosis due to severity of her multiple injuries. CT scan and upper GI series showed the diagnosis for the rest and allowed timely treatment.

All jejunal injuries except one were on the anti-mesenteric border, most of them were within 40 cm from the DJ flexure. All were treated surgically.

High complication rate in gastrointestinal perforation is usually due to delayed recognition. Grosfeld et al reported 35% complications with mortality rate of 12.5% [12].

Child abuse constitute a rare but an important cause for small bowel injury, it is reported in less than 0.5% of abused children, it is associated with high mortality rate of 45% [13] due to the nature of the presentation and the obscure circumstances that surround it [13]. Child abuse is an important cause of traumatic duodenal perforation in New Zealand a common presenting features are abdominal distension and vomiting [14]. Delayed recognition of intestinal injury related to child abuse is recognized to cause intestinal stricture [15].

Seatbelt marks in the restrained passenger involved in a road traffic accident are suggestive of gastrointestinal perforation, bowel wall injury, mesenteric injury and bladder injury [16,17].

Duodenal injuries are frequent following road traffic accidents and handle bar injuries, but full thickness perforation only happens in 15% of them [18], most of the injuries are in the 2^nd ^and 3^rd ^parts.

## Conclusion

Hollow visceral injuries are not very common sequels of abdominal injuries. Increasing number of duodenal wall hematoma is being recognized due to more liberal use of CT scan following blunt abdominal injury. All these were treated conservatively with no complications. Small bowel injuries mainly happen within close proximity to the DJ flexure and majority of them were located on the anti-mesenteric border. Most injuries were in males.

CT scan with continued clinical assessment is the best way to mange blunt abdominal trauma with suspected hollow visceral injuries.

**Table 1:** Summarises individual organ injuries, methods of diagnosis and management. CT scan was obtained using a spiral CT scanner with intravenous contrast.

Organ	Total	Diagnosis	Management	
			Conservative	Operative

Duodenum	10	CT (9); Upper GI series (1)	Haematoma (8)	Perforation (2)
Small Bowel	12	CT(5); X-ray (4); Clinical (3)		Perforation (11); Evisceration (1)
Large Bowel	6	CT (3); Clinical (3)	Haematoma (2)	Perforation (3); Haematoma (1)
Urinary Bladder	4	CT(4)	Extra peritoneal (2)	Intra peritoneal (2)

**Table 2:** Summarises mechanism of injury in different organs.

Mechanism of injury	No	Organs
MVA	17	Duodenal Haematoma (3), Small bowel (7), Colon (3), Bladder (4)
Handlebar	4	Duodenum (2) small bowel(2)
Fall from height	3	Duodenum (2) small bowel(1)
Hit by hard object	3	Duodenum (2) colon(1)
Horse kick	2	Duodenum (1) small bowel(1)
Stab wound	3	Colon (2) stomach (1)
Child abuse	1	Jejunal perforation

**Table 3:** Associated Injuries

Organ	No	Type of other injuries
Duodenum	6/10	Head injury (2), L1 compression (1) Splenic Rupture (1), Fractures (3)
Small bowel	1/12	Subdural haematoma (1)
Colon	1/6	Multiple fatal injuries (1)
Bladder	4/4	Pelvic fractures (3), Head injury (2) Splenic Rupture (1)
